# Staphylococcus capitis Endocarditis of a Native Valve

**DOI:** 10.7759/cureus.15738

**Published:** 2021-06-18

**Authors:** Steven Douedi, Mihir Odak, Andrew Ravin, Natasha Campbell

**Affiliations:** 1 Internal Medicine, Jersey Shore University Medical Center, Neptune City, USA

**Keywords:** staphylococcus capitis, infective endocarditis, native valve, mitral valve, opportunistic pathogen

## Abstract

Described as primarily an opportunistic pathogen, *Staphylococcus capitis* is primarily found as normal flora of the human skin but has been defined in literature as being a rare cause of infective endocarditis. We present a case of an otherwise healthy 65-year-old male who presented to our institution with symptoms similar to septic emboli. Blood cultures were obtained and ultimately grew *Staphylococcus capitis* in both bottles with repeat cultures one day later confirming the growth. A transthoracic echocardiogram was performed showing an ejection fraction of 60-65% and a thickened mitral value with mild-to-moderate mitral regurgitation. He was ultimately treated with IV cefazolin and improved with symptom resolution in outpatient follow-up. *Staphylococcus capitis* pathogenesis continues to be poorly understood, yet aggressive management with surgery and antibiotics has proven to decrease morbidity and mortality.

## Introduction

*Staphylococcus capitis* is a gram-positive coagulase-negative organism, which was first described in 1975 and was later found to be an uncommon cause of infective endocarditis (IE) in 1992 [1,2]. Few cases of *Staphylococcus capitis *endocarditis have been reported in the literature to date. Described as primarily an opportunistic pathogen, *Staphylococcus capitis* is primarily found as normal flora of the human skin, specifically on the scalp and forehead [3]. While *Staphylococcus capitis* has been defined in the literature as being a rare cause of IE, its pathogenesis continues to be poorly understood. Aggressive management with surgery and antibiotics has proven to decrease morbidity and mortality [3,4]. We present a case of sub-acute IE of the mitral valve with symptoms of septic emboli due to *Staphylococcus capitis* treated with IV antibiotics. Our hope in presenting this case is to encourage consideration for a broader group of organisms in patients who present with a similar constellation of symptoms and findings and to appropriately initiate prompt therapy.

## Case presentation

A 65-year-old male with no known medical history presented to the ED complaining of left great toe tenderness and erythema. He stated he was in his usual state of health until about one month prior to admission where he noted right knee swelling and pain that spontaneously improved. He denied any trauma or inciting causes at that time. Then, one week prior to this admission, he noted slurred speech and confusion, which prompted him to come to the ED. At that time neurological work-up with CT scan of the head was unremarkable and his symptoms resolved spontaneously within 4 hours of onset and he was discharged with outpatient follow-up. He stated that he then suddenly began to have left great toe pain, which again prompted him to come to the ED. He also endorsed a fever at home measuring 102 degrees Fahrenheit the day prior to admission but had no other complaints at that time.

In the ED he was noted to have a temperature of 98.8 degrees Fahrenheit, blood pressure of 144/63 mm Hg, heart rate of 78 beats per minute, and was saturating 100% on room air. Physical examination revealed a 3/6 holosystolic murmur at the apex with radiation to the left axilla, suggesting possible mitral regurgitation (MR) and mild erythema between the great and second toe of the left foot with tenderness to palpation of the webbed region between the toes. There were no splinter hemorrhages, Janeway lesions, or Osler nodes appreciated. Laboratory results were unremarkable on admission with a white blood cell count of 8.2 * 10^3^/µL (normal value: 4.5-11.0 * 10^3^/µL) and normal lactic acid and procalcitonin. Blood cultures were obtained and ultimately grew *Staphylococcus capitis* in both bottles. Repeat cultures were drawn one day after the initial cultures, which grew *Staphylococcus capitis *again, suggesting persistent bacteremia. A transthoracic echocardiogram was performed showing an ejection fraction of 60-65% and a thickened mitral value with moderate MR.

**Figure 1:**
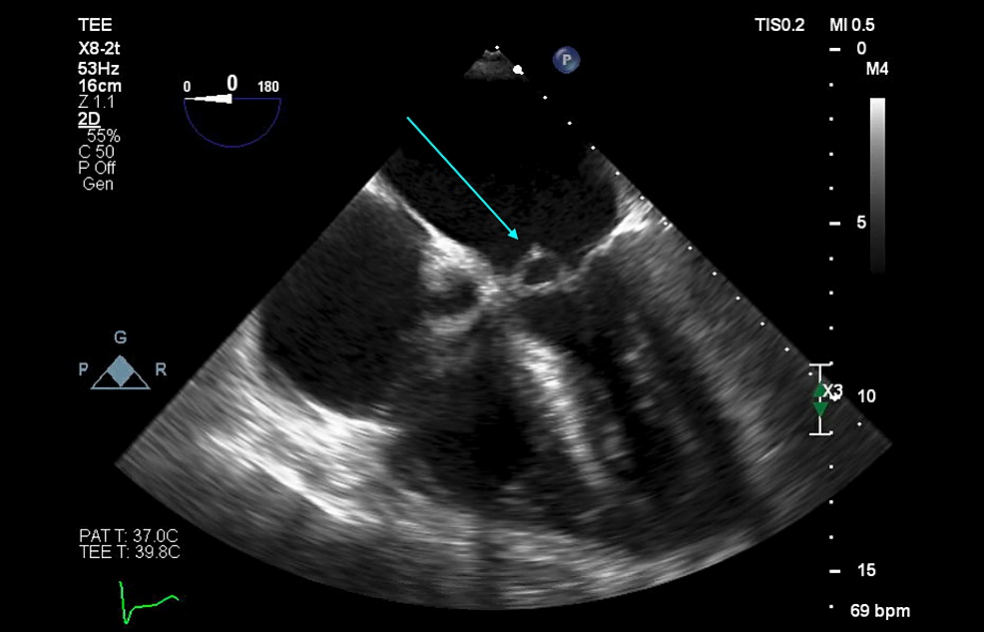
Mid-esophageal four-chamber view of transesophageal echocardiogram showing vegetation on the closed mitral valve (blue arrow)

**Figure 2:**
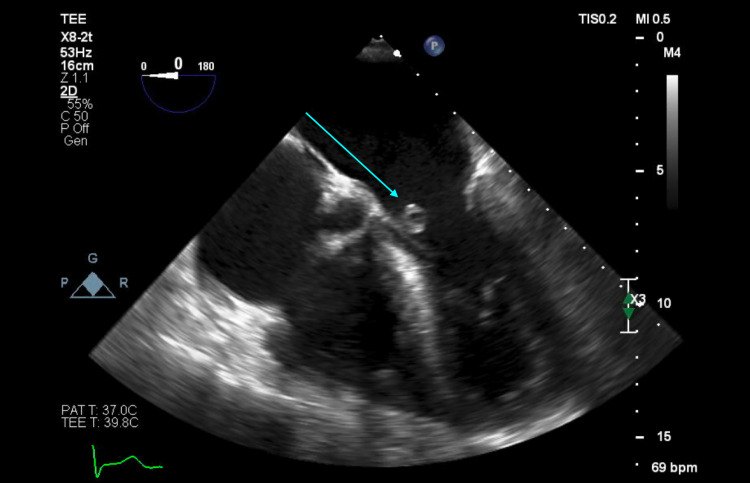
Mid-esophageal four-chamber view of transesophageal echocardiogram showing vegetation on an open mitral valve (blue arrow)

**Figure 3:**
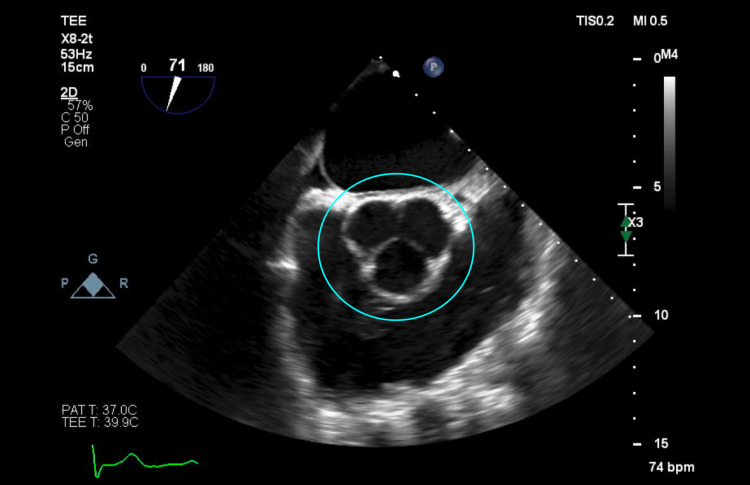
Mid-esophageal aortic valve short-axis view of transesophageal echocardiogram showing a tri-leaflet aortic valve with no vegetation and closed cusps (blue circle)

**Figure 4:**
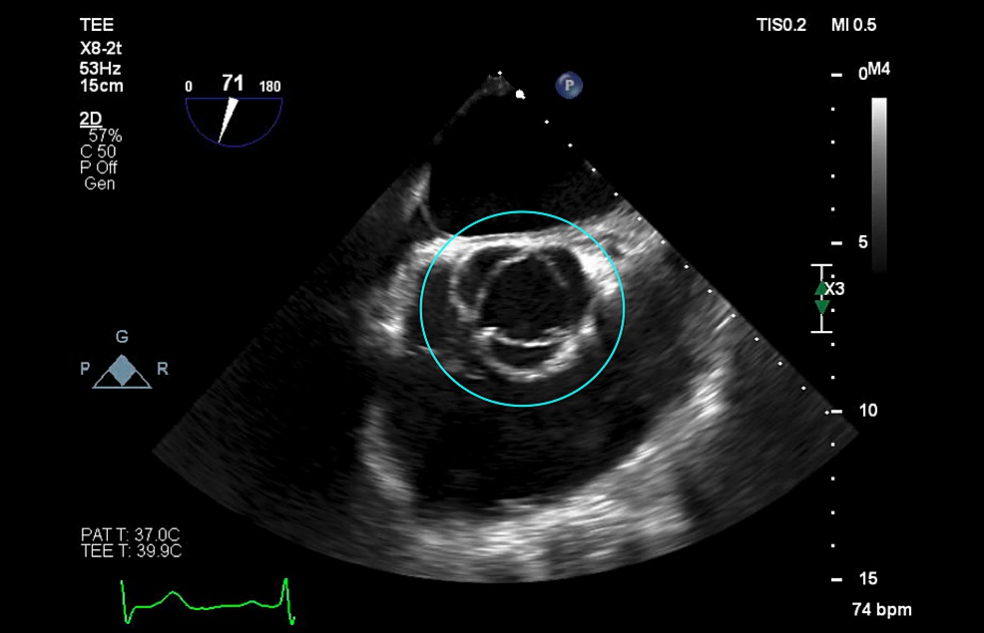
Mid-esophageal aortic valve short-axis view of transesophageal echocardiogram showing the aortic valve with no vegetation and open cusps (blue circle)

The patient was started on IV vancomycin 15 mg/kg every 12 hours and was planned to undergo a transesophageal echocardiogram (TEE). The TEE revealed a small, mobile density on the anterior leaflet of the mitral valve consistent with endocarditis, as confirmed by the cardiology team (Figures 1, 2), along with new-onset moderate MR. An examination of the aortic valve revealed a tri-leaflet valve with no vegetation (Figures 3, 4). The patient's persistent bacteremia along with positive echocardiographic findings of vegetation on the mitral valve, as confirmed by the cardiology team, satisfied two major criteria of the Modified Duke's Criteria, confirming definite IE. Antibiotics were adjusted to IV cefazolin 2 grams every 8 hours. After repeat blood cultures returned negative, the patient received a PICC line and was discharged on IV cefazolin 2 grams every 8 hours for a total of six weeks and with outpatient follow-up arrangements with the infectious disease and the internal medicine teams. At his one-month follow-up appointment, repeat blood cultures were noted to be negative, and he reported feeling well and symptom-free.

## Discussion

*Staphylococcus capitis* was first described in 1975 as a gram-positive coagulase-negative organism and has found to be a rare cause of IE [5]. IE, of any cause, has an approximate annual incidence of up to seven cases in 100,000 individuals [6]. Risk factors predisposing to IE include IV drug use, poor dental care, prosthetic valve implantation, and valvular disease [6]. IE is defined as an infection of the inner tissue layer of the heart, which most commonly includes the mitral followed by the aortic valves [7,8]. It usually results from bacteremia; circulating pathogens deposit on valves and form vegetations, which can then alter valve function, pump physiology, and can also result in atrioventricular block if infectious masses press on the conduction system [9,10].

Common pathogens that result in bacterial IE include *Staphylococcus aureus*, *Streptococcus viridians*, and HACEK organisms. Coagulase-negative *Staphylococcus* species are also frequently implicated; however, *Staphylococcus capitis* native valve endocarditis is extremely rare, with less than 15 cases reported to date since its identification in 1975 [8]. As *Staphylococcus​*​​​​​​* capitis* is a member of normal skin flora organisms, skin and soft tissue infections with *Staphylococcus capitis* are common [11]. In the previously reported cases of endocarditis, however, half occurred following procedural intervention, such as cardiac catheterization.

Our case is unique in that *Staphylococcus capitis* native valve IE is rarely reported in the literature. While the decision to pursue medical management or surgical correction for endocarditis depends on several factors, the appropriate antibiotic coverage and timely initiation depend on the organism responsible. Current guidelines by the American Association of Thoracic Surgery recommend surgical valve replacement in IE if valvular dysfunction results in heart failure, IE is caused by fungal or resistant organisms, IE induced heart block, aortic or annular abscess formation, or valvular penetration, or there is persistence of infection up to one week following antibiotic therapy, prosthetic valve IE, embolic phenomenon, or vegetations despite medical therapy [6]. As some of these indications rely on close observation of progress on medical therapy, prompt identification of the causative organism is imperative and antibiotics with appropriate coverage should be initiated immediately to reduce morbidity and mortality.

## Conclusions

*Staphylococcus capitis* native valve endocarditis is uncommon and underreported in the literature. While hospitalized patients are at great risk for resistant organisms, opportunistic pathogens also deserve consideration as such pathogens may cause significant damage to the heart. Treatment of *Staphylococcus capitis* IE should be promptly started with IV antibiotics and surgery may also be indicated to prevent complications and mortality.
